# Xanthogranulomatous Pyelonephritis with Incomplete Double Ureter

**DOI:** 10.1155/2017/2392670

**Published:** 2017-09-18

**Authors:** Yutaro Hayashi, Takashi Kawahara, Yusuke Hattori, Kota Shimokihara, Sohgo Tsutsumi, Daiji Takamoto, Taku Mochizuki, Jun-ichi Teranishi, Yasushi Yumura, Yasuhide Miyoshi, Masako Otani, Hiroji Uemura

**Affiliations:** ^1^Departments of Urology and Renal Transplantation, Yokohama City University Medical Center, Yokohama, Japan; ^2^Division of Diagnostic Pathology, Yokohama City University Medical Center, Yokohama, Japan

## Abstract

**Introduction:**

Xanthogranulomatous pyelonephritis (XGP) is a type of chronic renal inflammation that usually occurs in immunocompromised middle-aged women with chronic urinary tract infection or ureteral obstruction induced by the formation of ureteral stones. XGP with an incomplete double ureter is extremely rare.

**Case Presentation:**

A 76-year-old woman was referred to our department to undergo further examination for a left renal tumor that was detected by ultrasonography. Dynamic contrast computed tomography (CT) revealed an enhanced tumor in the upper renal parenchyma. Laparoscopic radical nephrectomy was performed based on a preoperative diagnosis of renal cell carcinoma. Histological sections showed the aggregation of foam cells; thus, XGP was diagnosed.

**Conclusion:**

We herein report a rare case of XGP in the upper pole of the kidney, which might have been associated with an incomplete double ureter.

## 1. Introduction

Xanthogranulomatous pyelonephritis (XGP) is a type of renal chronic inflammation [1]; it is histologically characterized by the invasion of lipid-laden macrophages (foam cells) into the renal parenchyma. XGP usually occurs in immunocompromised middle-aged women with chronic urinary tract infection or ureteral obstruction induced by the formation of ureteral stones. It is difficult to differentiate this entity from renal cell carcinoma on imaging. Due to the suspicion of renal cell carcinoma, many patients with focal XGP are subjected to nephrectomy.

XGP with incomplete double ureter is extremely rare; furthermore, focal XGP is difficult to diagnose preoperatively. We herein report a case of XGP with incomplete double ureter.

## 2. Case Presentation

A 76-year-old female was referred to our department for the further examination of her left renal tumor. Ultrasonography was performed by her previous clinic in order to investigate slight back pain. She had no remarkable history other than repeated pyelonephritis. No further urological examinations were performed to investigate the cause of her repeated pyelonephritis. Her mother had a history of renal cell carcinoma. The laboratory data showed almost normal findings, with the exception of slight CRP elevation (1.04 mg/dL).

**Figure 1:**
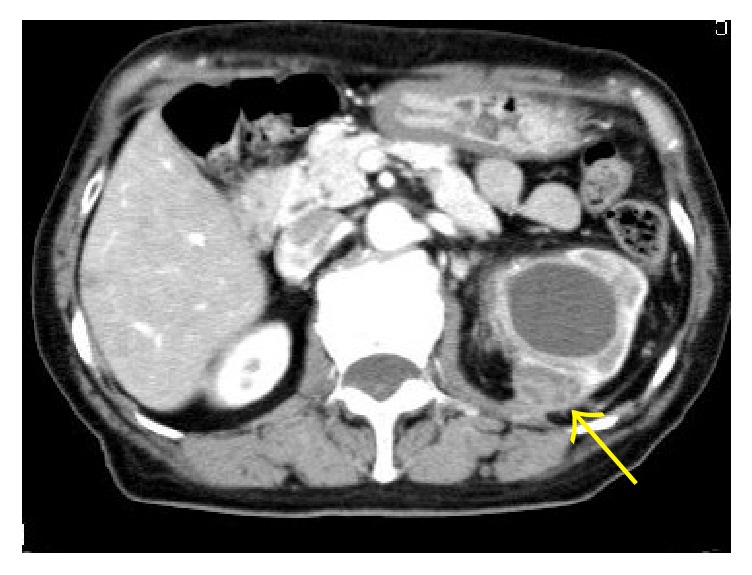
CT image: left renal mass with contrasted surface (arrow).

**Figure 2:**
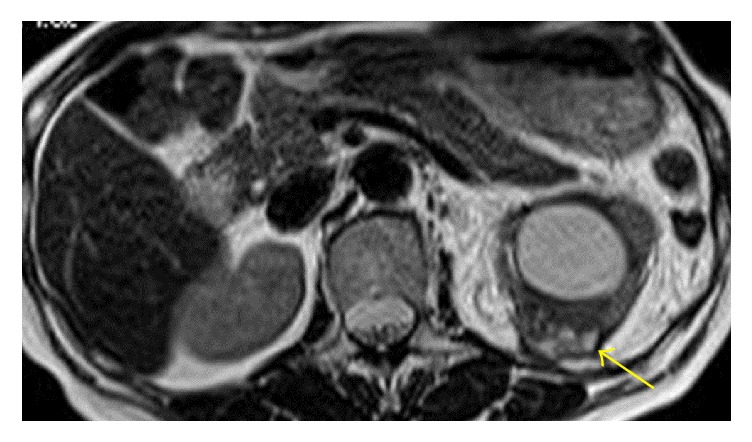
MRI: the tumor contained small cysts (arrow).

**Figure 3:**
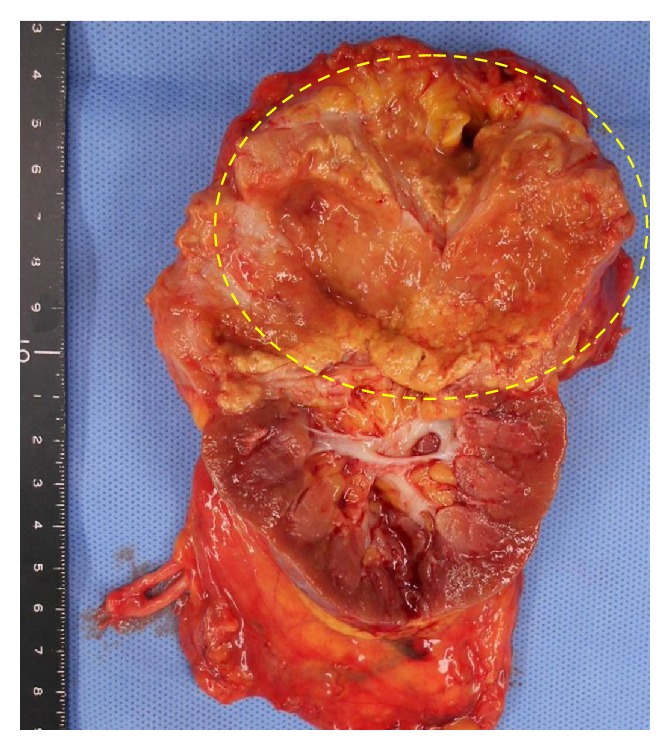
The resected specimens: a yellowish-white tumor in the upper renal parenchyma and incomplete double ureters.

Dynamic contrast computed tomography (CT) revealed an enhanced tumor of 3 cm in diameter in the upper renal parenchyma (Figure 1). At that time, we had not performed any other examinations to detect urological abnormalities, because the upper renal lesion was thought to be a cyst, not hydronephrosis. Magnetic resonance imaging (T2-weighted imaging) detected a small, high-density cyst (Figure 2). Due to the perioperative diagnosis of renal cell carcinoma, laparoscopic radical nephrectomy was performed. The tumor adhered strongly to the muscular fascia of the lumbar quadrant; thus, they were resected together. The resected kidney showed a yellowish-white tumor in the upper parenchyma and incomplete double ureters (Figure 3). Purulent liquid from the tumor was found to contain* Escherichia coli*. She was discharged from the hospital on the seventh day after the operation.

**Figure 4:**
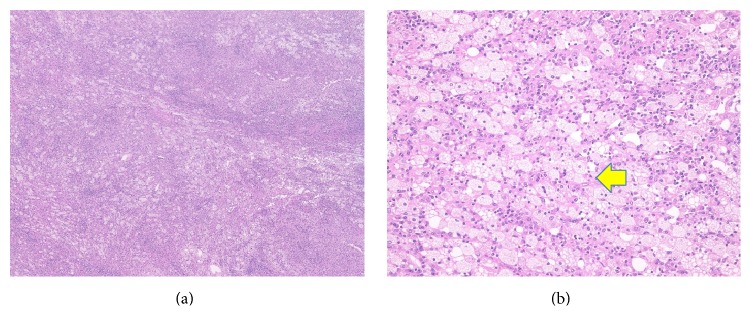
Infiltration of inflammatory cells including foam cells, plasma cells, and lymphocytes ((a) ×40). There is aggregation of foam cells (arrow) ((b) ×400) in hematoxylin and eosin stain.

Histological specimens showed the aggregation of foam cells; plasma cells, lymphocytes, and neutrophils were also noted. Based on the histological findings, the patient was diagnosed with XGP (Figure 4).

## 3. Discussion

XGP is a type of chronic renal inflammation. Pathologically, lipid-filled macrophages (foam cells) invade the renal parenchyma [2]. XGP usually occurs in immunocompromised middle-aged females with chronic urinary tract infection or ureteral obstruction induced by the formation of ureteral stones [1]. The chief complaints include fever, flank pain, fatigue, and body weight loss. Laboratory examinations usually show an elevated white blood cell count and anemia [1]. Urine cultures are positive in 70% of cases, usually for proteus species and* E. coli* [1]. As our patient had an incomplete double ureter, ureteral stenosis might have resulted in chronic urinary tract infection, thereby causing XGP.

Despite the development of advanced CT, MRI, and ultrasonography (US) techniques, it remains difficult to determine whether or not a tumor is malignant. XGP typically appears as a low-echoic lesion on US, while CT shows a weakly enhanced tumor surrounded by a highly enhanced surface [1, 3, 4]. However, despite these findings, the preoperative diagnosis of XGP remains difficult. MRI fat suppression imaging has been reported to be useful, but angiomyolipoma, retroperitoneal liposarcoma, and some renal cell carcinomas also contain fat lesions; thus the preoperative diagnosis of XGP remains difficult [1, 5].

XGP can be divided into diffuse and focal types based on its radiographic features. More than 80% of cases are reported to be the diffuse type. In this case, XGP was detected locally in the renal parenchyma; thus this case was classified as focal-type XGP. XGP shows specific findings of large, clear foam cells. The rapid diagnosis of frozen pathological sections has been attempted in previous studies; however, foam cells appear similar to the cells in renal clear cell carcinoma; thus, a differential diagnosis was found to be difficult. On immunohistochemical analyses, XGP is positive for CD68 and negative for EMA and CD10 [6]. Focal XGP is usually difficult to differentiate from renal cell carcinoma preoperatively and surgical resection is usually required for a diagnosis.

## 4. Conclusion

In conclusion, we encountered a rare case of XGP in the upper pole of the kidney with an incomplete double ureter.

## Additional Points

*Availability of Data and Material*. Due to ethical restrictions, the raw data underlying this paper are available from the corresponding author upon request.
